# Comparative Population Genetic Structure of the Endangered Southern Brown Bandicoot, *Isoodon obesulus*, in Fragmented Landscapes of Southern Australia

**DOI:** 10.1371/journal.pone.0152850

**Published:** 2016-04-20

**Authors:** You Li, Steven J. B. Cooper, Melanie L. Lancaster, Jasmin G. Packer, Susan M. Carthew

**Affiliations:** 1 School of Biological Sciences, the University of Adelaide, Adelaide, SA, 5005, Australia; 2 Australian Centre for Evolutionary Biology and Biodiversity, the University of Adelaide, Adelaide, SA, 5005, Australia; 3 Northwest University for Nationalities, Lanzhou, Gansu, 730030, China; 4 Evolutionary Biology Unit, South Australian Museum, North Terrace, Adelaide, SA, 5000, Australia; 5 Research Institute for Environment and Livelihoods, Charles Darwin University, Darwin, NT, 0909, Australia; Chinese Academy of Sciences, CHINA

## Abstract

Genetic connectivity is a key factor for maintaining the persistence of populations in fragmented landscapes. In highly modified landscapes such us peri-urban areas, organisms’ dispersal among fragmented habitat patches can be reduced due to the surrounding matrix, leading to subsequent decreased gene flow and increased potential extinction risk in isolated sub-populations. However, few studies have compared within species how dispersal/gene flow varies between regions and among different forms of matrix that might be encountered. In the current study, we investigated gene flow and dispersal in an endangered marsupial, the southern brown bandicoot (*Isoodon obesulus*) in a heavily modified peri-urban landscape in South Australia, Australia. We used 14 microsatellite markers to genotype 254 individuals which were sampled from 15 sites. Analyses revealed significant genetic structure. Our analyses also indicated that dispersal was mostly limited to neighbouring sites. Comparisons of these results with analyses of a different population of the same species revealed that gene flow/dispersal was more limited in this peri-urban landscape than in a pine plantation landscape approximately 400 km to the south-east. These findings increase our understanding of how the nature of fragmentation can lead to profound differences in levels of genetic connectivity among populations of the same species.

## Introduction

Habitat loss and fragmentation are the leading threats to biological diversity worldwide [1, 2], and the rapid spread of urbanisation is a major driver of landscape degradation and fragmentation. In urban landscapes, once-continuous habitat is largely being replaced with fragmented remnants surrounded by a heterogeneous matrix of variable human constructs including buildings, roads, parks, gardens and even agricultural land in some peri- or semi-urban areas.

The importance of different types of matrices in fragmented landscapes has been recognised and their influences on biodiversity (e.g. isolation effects, being alternative or secondary habitats, and providing corridors and stepping stones) have been investigated in numerous studies (see review by Prevedello & Vieira [3]). In highly modified landscapes with complex matrices, movements of organisms and subsequent gene flow between habitat patches can be limited by landscape features such as roads, rivers or other matrices with unsuitable habitat (e.g. [4–6]). Reduced gene flow can lead to a range of consequences for the remnant populations, including increased inbreeding, loss of genetic diversity through genetic drift and the potential for increased extinction risk [7, 8]. Thus, the degree to which organisms are able to move across a heterogeneous matrix is crucial for the persistence of populations in fragmented landscapes [9–12].

In practice, the effects of matrices on organisms’ movements between habitat patches are species-specific [3, 12], and dispersal capacity and genetic structure of species can vary depending on the type of matrix they encounter, their demographic history and their geographical location (e.g. within species, populations at lower latitudes tend to have greater genetic divergence than populations at higher latitudes; [13]) [14]. Although, there is now a large body of work on gene flow and dispersal capability for natural and anthropogenically modified landscapes (e.g. [5, 15–19]), few studies have investigated how different forms of fragmentation with distinct matrices influence patterns of population connectivity within the same species (but see [20, 21]). Such studies are useful because they improve our understanding of dispersal dynamics and help with decision-making for conservation management of threatened species (e.g. identifying priority areas for habitat restoration and approaches for developing habitat corridors).

Populations of the southern brown bandicoot species (*Isoodon obesulus*) in South Australia (SA) and their habitat represent an ideal system to explore this issue. *I*. *obesulus* is a rabbit-sized ground-dwelling marsupial, which has dramatically declined in number over the last 220 years, with studies providing evidence for a contracted distribution and local population extinctions [22–25]. In South Australia, *I*. *obesulus* is the only surviving member of the family Peramelidae. This family originally included eleven Australian species before European settlement, but now only eight species survive in the whole country. The subspecies *I*. *o*. *obesulus*, distributed in eastern and southern regions of Australia (including SA), is listed as Nationally Endangered (Australian Environment Protection and Biodiversity Conservation Act 1999), and, therefore, further understanding of the impact of habitat fragmentation on population connectivity has important implications for conservation management.

**Fig 1:**
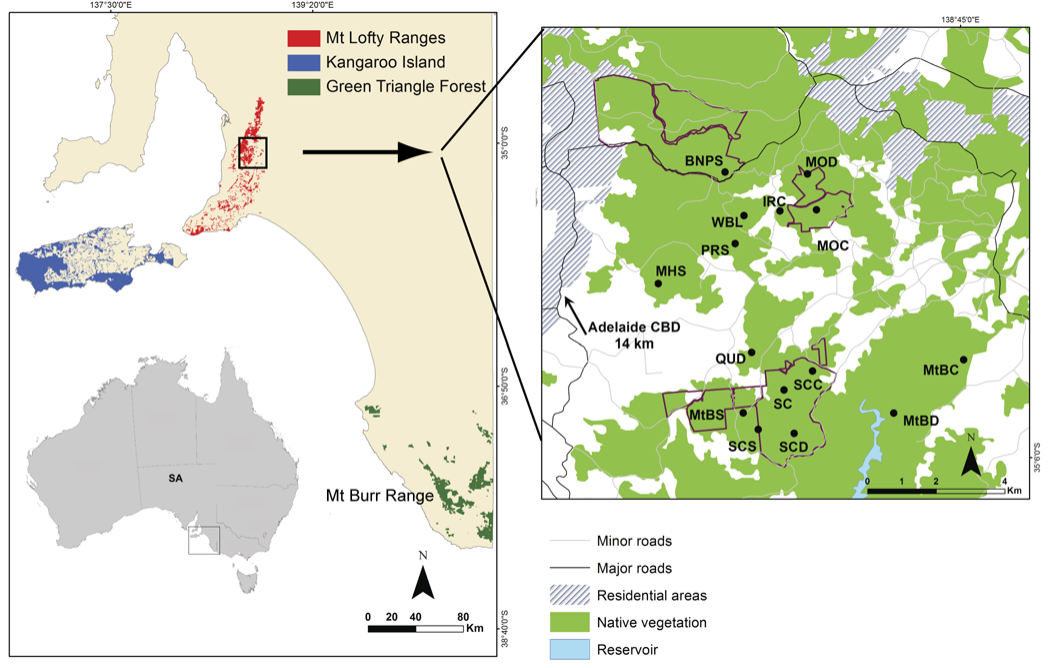
Map of the location of three strongholds of *I*. *obesulus* in South Australia (SA), Australia (left hand side), with the box indicating the location of sites in the central Mount Lofty Ranges that were used in the current study. Cleared lands are represented in white and the borders of the national parks are represented in purple. See Table 1 for full names of the sampled sites.

Three current strongholds of *I*. *obesulus* persist in South Australia–the Mount Lofty Ranges, Kangaroo Island and the south-east region (Fig 1). Within the latter region, *I*. *obesulus* occurs only in the Green Triangle Forest area (comprised of three forest districts–Mount Burr, Mount Gambier and Penola), one of Australia’s major softwood plantation regions currently managed by the state government-based organisation, ForestrySA. Here, numerous small fragments of native forest are managed as reserves embedded in matrices of pine (*Pinus radiata*) plantations or agricultural land. Investigation of genetic connectivity of *I*. *obesulus* showed significant population genetic structuring and restricted gene flow and dispersal to neighbouring patches [26].

The Mount Lofty Ranges are another stronghold of *I*. *obesulus*. With its rich biodiversity, the area was identified as one of 15 Australian biodiversity hotspots by the Commonwealth Government in 2003 (Department of the Environment, National Biodiversity Hotspots, http://www.environment.gov.au/biodiversity/conservation/hotspots/national-biodiversity-hotspots). However, the region has experienced extensive native vegetation clearance and only 13% of the original vegetation remains [27]. It is also highly fragmented with few relatively intact areas and variable amounts of degraded native vegetation embedded in a heterogeneous matrix of urban and agricultural land uses.

This study aimed to investigate population structure, genetic diversity and the extent of gene flow of *I*. *obesulus* in the central Mount Lofty Ranges (Fig 1 and Table 1), and test the hypothesis that the species here exhibits genetic structuring due to reduced gene flow across the fragmented landscape. In addition, since the habitat and matrix structure of the Mount Lofty Ranges is different and seemingly less fragmented than the previously studied Mount Burr Range [26], results obtained here were then compared to the Mount Burr study to discuss connectivity of bandicoot populations in different matrix systems.

**Table 1:** Sampling information and genetic diversity parameters for *I*. *obesulus* at 15 sites within the Mount Lofty Ranges.

Site name	Site abbreviation	Distance to nearest sampled site* (km)	*N*	*H*_*O*_	*H*_*E*_	*F*_*IS*_	*A*	*AR*	*IR*
Belair National Park	BNPS	1.39	40	0.381	0.400	0.047	2.29	1.620	0.472
Ackland Hill Rd Coromandel (Mud Hut)	MHS	2.53	37	0.506	0.499	-0.014	3.64	1.959	0.236
Pole Rd	PRS	0.92	13	0.412	0.501	0.187	3.14	2.031	0.270
Wirra Birra low	WBL	0.92	11	0.522	0.542	0.039	2.86	2.038	0.185
Ironbank Rd	IRC	1.07	15	0.464	0.550	0.163	3.50	2.017	0.310
Mark Oliphant CP Site 1	MOD	1.08	15	0.537	0.552	0.028	2.93	1.976	0.175
Mark Oliphant CP Site 2	MOC	1.07	22	0.475	0.550	0.141	3.14	2.041	0.234
Dorset Vale Road	QUD	1.45	24	0.476	0.569	0.167	4.14	2.208	0.222
Mount Bold Reserve Site 1	MtBS	0.63	17	0.414	0.480	0.143	3.50	1.941	0.345
Scott Creek CP Site 1	SCS	0.63	7	0.594	0.598	0.011	2.93	2.208	0.050
Scott Creek CP Site 2	SC	0.97	12	0.383	0.581	**0.352**	3.86	2.272	0.389
Scott Creek CP Site 3	SCC	0.97	7	0.709	0.538	**-0.354**	2.86	2.026	-0.116
Scott Creek CP Site 4	SCD	1.05	6	0.599	0.627	0.048	3.07	2.347	0.102
Mount Bold Reserve Site 2	MtBC	2.59	12	0.446	0.520	0.147	3.29	2.084	0.263
Mount Bold Reserve Site 3	MtBD	2.59	16	0.524	0.575	0.094	3.36	2.129	0.203

Sample size (*N*), observed heterozygosity (*H*_*O*_), expected heterozygosity (*H*_*E*_), inbreeding coefficient (*F*_*IS*_), allelic diversity (*A*), allelic richness (*AR*), and internal relatedness (*IR*). Significant *F*_*IS*_ values were denoted in bold (*P* < 0.05). CP = Conservation Park

* The “distance to nearest sampled site” was measured in ArcGIS 10 as straight-line distance.

## Methods

### Study area

We surveyed 15 sites within the distribution of *I*. *obesulus* in the central Mount Lofty Ranges, with seven sites located in three national/conservation parks (Fig 1 and Table 1). Due to a lack of information on the density or level of coverage of native vegetation in the Mount Lofty Ranges, Fig 1 shows only the broad distribution of native vegetation in this region. Suitable bandicoot habitats in this region are patchily distributed and often surrounded by open areas with very low cover of native vegetation, even within the conservation park (e.g. Scott Creek Conservation Park). These open areas make it unlikely that any of our adjacent collection sites in the Mount Lofty Ranges are connected by continuous habitat. These national/conservation parks were thus not treated as continuous habitat.

Trapping sites were chosen based on our own field observations of fresh bandicoot diggings (conical holes produced during foraging; [28]) and vegetation characteristics–the species is known to be associated with the grass tree *Xanthorrhoea australis* [28]. Within sites, traps were set as two 200 m-long parallel transects of 10 cage traps (55 × 25 × 25 cm treadle) and 10 Elliott traps (330 × 100 × 100 mm) per transect. Both traps were placed on the ground, covered with hessian or plastic bags to protect animals from rain and sun, bedded with small pieces of hessian to keep animals warm, and baited with a mixture of peanut butter and oats. Traps were set and checked in the morning and late afternoon during summer, autumn and spring and in the morning during winter. Captured bandicoots were processed on site or nearby. Individuals were permanently identified with a passive integrated transponder tag inserted subcutaneously in the rump above the right hind leg [29]. A small notch of skin was removed from the ear for secondary identification and stored in a 50: 50 solution containing ethanol and saline for genetic analysis. A total of 284 bandicoot tissue samples were collected during 11 field trips between 2008 and 2011. Of the 284 samples, 30 were collected from pouch young. These samples were excluded and 254 samples were used in the population analyses.

### DNA extraction and genotyping

DNA was isolated using the Gentra Puregene extraction kit, following the manufacturer’s instructions (Gentra Systems Inc.). Individuals were genotyped at 14 microsatellite loci developed for *I*. *obesulus*: five (B3-2, B15-1, B20-5, B34-2, and B38-1) by Zenger & Johnston [30], and nine by Li et al. [31]. PCR amplifications followed protocols in Li et al. [31]. Approximately 10% of samples were genotyped twice at all loci to check error rates. These were expressed as the number of errors per allele, which was calculated as the number of incorrect alleles divided by the total number of genotyped alleles [32]. Repeat genotyping error rate was very low, with an average of 0.0009 across all loci. Amplified products were run on an ABI 3730 DNA Analyser and alleles were scored using GeneMapper 4.0 (Applied Biosystems).

### Microsatellite analyses

Variation in allele frequencies within sites sampled across multiple years was examined via ANOVA [33] in SPSS v.20 (SPSS Inc., Chicago, IL, USA). Since no significant variation in allele frequencies across years was found for any of the 11 sites examined we were confident about pooling data across years.

For this study, we did not have complete information on the juvenile or adult status of each individual. Previous analyses of the Mount Burr population [26] indicated that juveniles are capable of dispersal and their inclusion in population analyses did not affect the results obtained. However, to address potential concerns of sampling bias due to the presence of related individuals, we used COANCESTRY v.1.0.1.2 [34] to investigate the level of genetic relatedness between individuals using a triadic likelihood estimator (TrioML) [35].

Conformation with Hardy-Weinberg equilibrium (HWE) across loci and sites was assessed in Genepop 4.1.0 [36] and a test for linkage disequilibrium among loci conducted in Arlequin 3.11 [37]. MICRO-CHECKER v.2.2.3 [38] was used to estimate null allele frequencies. Sequential Bonferroni corrections [39] were applied to adjust significance values for multiple comparisons.

Number of alleles per locus (*A*), observed heterozygosity (*H*_*O*_) and expected heterozygosity (*H*_*E*_) were calculated in Arlequin 3.11 [37], and inbreeding coefficient (*F*_*IS*_) and allelic richness (*AR*, corrected for sample size) were estimated in FSTAT 2.9.3.2 [40]. An estimate of parental relatedness was calculated using internal relatedness (*IR*) [41] in an R extension package, Rhh [42].

### Genetic differentiation and population structure

To assess the degree of genetic differentiation of bandicoots across sites, we measured pairwise *F*_*ST*_ in Arlequin 3.11 [37] and calculated pairwise *D*_*EST*_ as a measurement of actual differentiation in the package DEMEtics [43] implemented in R, with 1 000 bootstrap iterations to determine statistical significance. *D*_*EST*_ takes account of the effective number of alleles and may perform better than *F*_*ST*_ in the case of highly polymorphic markers such as microsatellites [44].

We implemented Bayesian clustering analysis in STRUCTURE 2.3.3 [45] and TESS 2.3.1 [46, 47] to characterize population genetic structure. STRUCTURE uses a non-spatial Bayesian algorithm, while TESS incorporates spatial information into the analysis and thus increases the power of modelling genetic structure [48]. STRUCTURE analysis used an admixture model with correlated allele frequencies, a burn-in of 100 000 and 100 000 MCMC steps after the burn-in. The value of *K* (*K* is the number of likely clusters) was set from 1 to 15 with ten replicates of each *K* to verify the convergence of the Markov chain. The method described in [49] was used for determining the most likely *K*. For TESS, we ran the analysis under an admixture model using *K* ranging from 2 to 15 (10 replicates per *K*), with 10 000 burn-in and 50 000 sweeps. The value of the interaction parameter ψ (the strength of the spatial autocorrelation) was set to the default value, 0.6. The optimal *K* for TESS was chosen as the one with the stabilized value of the Deviance Information Criterion (DIC). For both analyses, CLUMPP 1.1.2 [50] was used to average the membership probabilities for the ten runs of the most likely *K* and DISTRUCT 1.1 [51] was used to display the averaged results.

### Spatial scale of genetic differentiation

To investigate the effect of isolation by distance (IBD), we ran Mantel tests (at both individual and site level) between linearised genetic distance (*F*_*ST*_ / (1-*F*_*ST*_) and *D*_*EST*_ / (1- *D*_*EST*_)) and the logarithm of geographical distance using the subprogram Isolde of Genepop 4.1.0 [36] with 10 000 permutations.

To complement the IBD analyses, we also conducted a redundancy analysis (RDA) to explore the relationship between genetic structure and explanatory variables (see below). RDA is an analogue of multivariate linear regression and is reported to have greater power than Mantel tests in cases where there are multivariate species-environment relationships [52]. We used allele frequencies as dependent variables (unconstrained matrix) and environmental variables as independent variables (constrained matrix). The latter included geographic coordinates, minimum distance to neighbouring site (DN) and degree of isolation (DI). The degree of isolation for each site was measured as the mean distance to the closest three sites. Partial RDA analyses, in which genetic variance was conditioned on the remaining variables, were conducted for any significant associations between genetic variance and one or more environmental variables. Partial and the full model with all explanatory variables also allowed us to examine how much of the genetic variance was uniquely explained by each variable, and how much was due to the joint effect of all the variables [53]. RDA analyses were conducted in the R package vegan [54].

Spatial autocorrelation analyses were performed in GenAlEx 6.41 [55] to further study the spatial scale of genetic variation. We used 0.5 km for distance class size, using separate analyses for males (*n* = 140) and females (*n* = 105) to check for sex-biased dispersal (nine samples of unknown gender were excluded). Statistical testing was based on the 95% confidence interval defined by 1 000 random permutations.

### Migration and gene flow among populations

We explored for recent migration rates among both sites and STRUCTURE clusters with the program BayesAss3.0.3 [56]. BayesAss uses a Bayesian MCMC approach to estimate asymmetric migration over the last two to three generations [56]. After a few preliminary runs to ensure convergence of the MCMC analysis, a chain length of 30 million iterations with a burn-in of 3 million iterations and a thinning interval of 2 000 was chosen to run the program. Various delta values (delta is the parameter that defines the size of the proposed change to the parameter values at each iteration) were used for migration rates, allele frequencies and inbreeding values. Multiple runs were performed with unique random seeds to assess convergence.

Migrants and individuals with mixed ancestry were detected in both STRUCTURE 2.3.3 [45] and GENECLASS 2.0 [57]. In STRUCTURE, the analysis was performed using sampling locations (i.e. sites) as prior population information with 100 000 burn-in and 100 000 MCMC steps after the burn-in. In GENECLASS, the test of first-generation migrants was performed using a Bayesian approach [58] and the Monte Carlo re-sampling method of Paetkau et al. (2004) [59] with 10 000 simulated individuals and an alpha of 0.05. For the likelihood computation, we used the likelihood ratio L_home (the likelihood of a given individual being from the population where it was sampled) because it is more appropriate than other estimations if not all source populations were sampled [59].

### Ethics statement

The protocol of sample collections in this study was performed under the University of Adelaide Animal Ethics Committee (project number S-2011-041) and Department of the Environment, Water and Natural Resources (DEWNR) permit to undertake scientific research (permit number G23771-13).

## Results

### Genetic variability

Results from COANCESTRY indicated that sampling was not biased towards highly related individuals. Mean relatedness (*R*) was 0.08, ranging from 0.04 in SC to 0.19 in SCC. Site SC had the fewest (*R* ≥ 0.5, *n* = 0) and BNPS had the most highly related individuals (*R* ≥ 0.5, *n* = 69) (*n* = the number of within-site pairwise relatedness comparisons where *R* was found to be ≥ 0.5, total number of comparisons were 780 for site BNPS and 66 for SC). We repeated the analyses with the related individuals excluded. Since their exclusion did not alter results of the population analyses (data not shown) we retained the entire data set for analyses.

Significant linkage disequilibrium was detected in 18 of the 1365 (1.3%) pairwise locus combinations and none of these were consistent across sites. Close physical linkage between any of the 14 loci was therefore considered unlikely. Twenty-one of the 210 locus × sites tests departed significantly from HWE after sequential Bonferroni correction, involving six loci (Ioo7, Ioo5, Ioo4, Ioo3, Ioo2, B15-1, B20-5 and B3-2). Three loci (Ioo7, Ioo3 and B3-2) deviated from HWE at more than two sites. Micro-checker detected that these three loci might contain null alleles. However, the presence of null alleles at these loci was not consistent across sites. In addition, deviations from HWE can result from inbreeding or the Wahlund effect (the reduction of heterozygosity due to population subdivision). To be cautious, we ran all the analyses without the three loci and the results showed a similar pattern to that of the full 14 locus data set. For this reason, we did not apply a correction for null alleles and retained the three loci for the analyses presented here.

Numbers of alleles per locus ranged from 3 (Ioo6, Ioo16, B20-5, and B38-1) to 10 (Ioo4, Ioo5, and B15-1) with an average of 6.1. At each site, mean observed heterozygosity across loci ranged from 0.381 (site BNPS) to 0.709 (site SCC) and expected heterozygosity ranged from 0.400 (BNPS) to 0.627 (SCD) (see Table 1 for site codes and heterozygosity values). *F*_*IS*_ values ranged from -0.354 in SCC to 0.352 in SC (Table 1). Allelic diversity (the average number of alleles per locus, *A*) was lowest in BNPS (2.29) and highest in QUD (4.14), with a mean of 3.23 (Table 1). Allelic richness ranged from 1.620 (BNPS) to 2.347 (SCD) (Table 1). Statistical tests showed no evidence of variation in *H*_*O*_, *H*_*E*_ and *AR* among all sites (ANOVA, F (14, 195) = 1.616, 1.796, and 1.464, *P* = 0.077, 0.071 and 0.128 respectively). Internal relatedness (*IR*) was highest in BNPS (0.472) and lowest in SCC (-0.116) (Table 1). Post hoc tests showed that bandicoots in BNPS had significantly higher values of *IR* than those from ten other sites (see S1 Table for details). *IR* in SCC was significantly lower than ten other sites (S1 Table).

### Genetic population structure and gene flow

Pairwise *F*_*ST*_ values were significant for 96 of the 105 comparisons (Table 2). The highest pairwise *F*_*ST*_ was between BNPS and MtBS (*F*_*ST*_ = 0.422, *P* < 0.05), and the lowest was between SCC and SCD (*F*_*ST*_ = 0.005, *P* = 0.265). Pairwise *D*_*EST*_ values were generally higher than *F*_*ST*_, with the highest value between BNPS and MHS (*D*_*EST*_ = 0.473, *P* < 0.05) and the lowest between SCC and SCD (*D*_*EST*_ = 0.024, *P* = 0.587). Mantel tests showed a significant association between genetic distance and geographical distance (measured as logarithm of (1 + geographical distance)) at both individual and site level (individual level: â statistic, *P* < 0.05; site level: *F*_*ST*_, *P* < 0.05, *D*_*EST*_, *P* < 0.05).

**Table 2:** Pairwise *F*_*ST*_ values (below diagonal) and pairwise *D*_*EST*_ values (above diagonal) estimated for the 15 sites sampled for *I*. *obesulus* (following Sequential Bonferroni correction).

DEST	BNPS	MHS	PRS	WBL	IRC	MOD	MOC	QUD	MtBS	SCS	SC	SCC	SCD	MtBC	MtBD
BNPS	-	0.473*	0.315*	0.193*	0.433*	0.373*	0.350*	0.415*	0.446*	0.309*	0.400*	0.357*	0.354*	0.378*	0.356*
MHS	0.410*	-	0.184*	0.347*	0.229*	0.194*	0.141*	0.221*	0.352*	0.288*	0.329*	0.300*	0.192*	0.249*	0.378*
PRS	0.361*	0.095*	-	0.153*	0.187*	0.191*	0.117*	0.328*	0.399*	0.316*	0.322*	0.357*	0.289^NA^	0.159*	0.318*
WBL	0.267*	0.211*	0.109*	-	0.163*	0.215*	0.211*	0.330*	0.346*	0.262*	0.266*	0.346*	0.306*	0.260*	0.355*
IRC	0.413*	0.174*	0.107*	0.092*	-	0.160^NA^	0.190*	0.247*	0.319*	0.441^NA^	0.318*	0.318*	0.305^NA^	0.257*	0.429*
MOD	0.326*	0.166*	0.108*	0.085*	0.130*	-	0.076*	0.251*	0.326*	0.261^NA^	0.361*	0.294*	0.232^NA^	0.238*	0.275*
MOC	0.339*	0.115*	0.065*	0.123*	0.155*	0.055*	-	0.307*	0.364*	0.260*	0.354*	0.317*	0.239^NA^	0.213*	0.300*
QUD	0.359*	0.167*	0.198*	0.190*	0.184*	0.151*	0.210*	-	0.109*	0.184*	0.154*	0.133*	0.056	0.237*	0.230*
MtBS	0.422*	0.244*	0.266*	0.210*	0.229*	0.194*	0.256*	0.059*	-	0.229*	0.114*	0.129*	0.127^NA^	0.282*	0.289*
SCS	0.361*	0.214*	0.204*	0.192*	0.284*	0.125*	0.159*	0.132*	0.217*	-	0.181*	0.161*	0.041^NA^	0.225*	0.172*
SC	0.395*	0.178*	0.207*	0.173*	0.188*	0.178*	0.216*	0.041	0.044	0.127*	-	0.154*	0.083	0.228*	0.307*
SCC	0.319*	0.203*	0.215*	0.173*	0.233*	0.164*	0.182*	0.081*	0.098*	0.105*	0.060	-	0.024	0.289*	0.180*
SCD	0.325*	0.130*	0.156*	0.124*	0.167*	0.082	0.123*	0.016	0.100*	0.054	0.029	0.005	-	0.254*	0.121*
MtBC	0.407*	0.141*	0.094*	0.205*	0.183*	0.148*	0.126*	0.150*	0.212*	0.160*	0.140*	0.176*	0.152*	-	0.274*
MtBD	0.301*	0.194*	0.172*	0.200*	0.258*	0.114*	0.135*	0.143*	0.219*	0.083*	0.187*	0.120*	0.077	0.152*	-

*0.05 significance level

NA = not available

**Fig 2:**
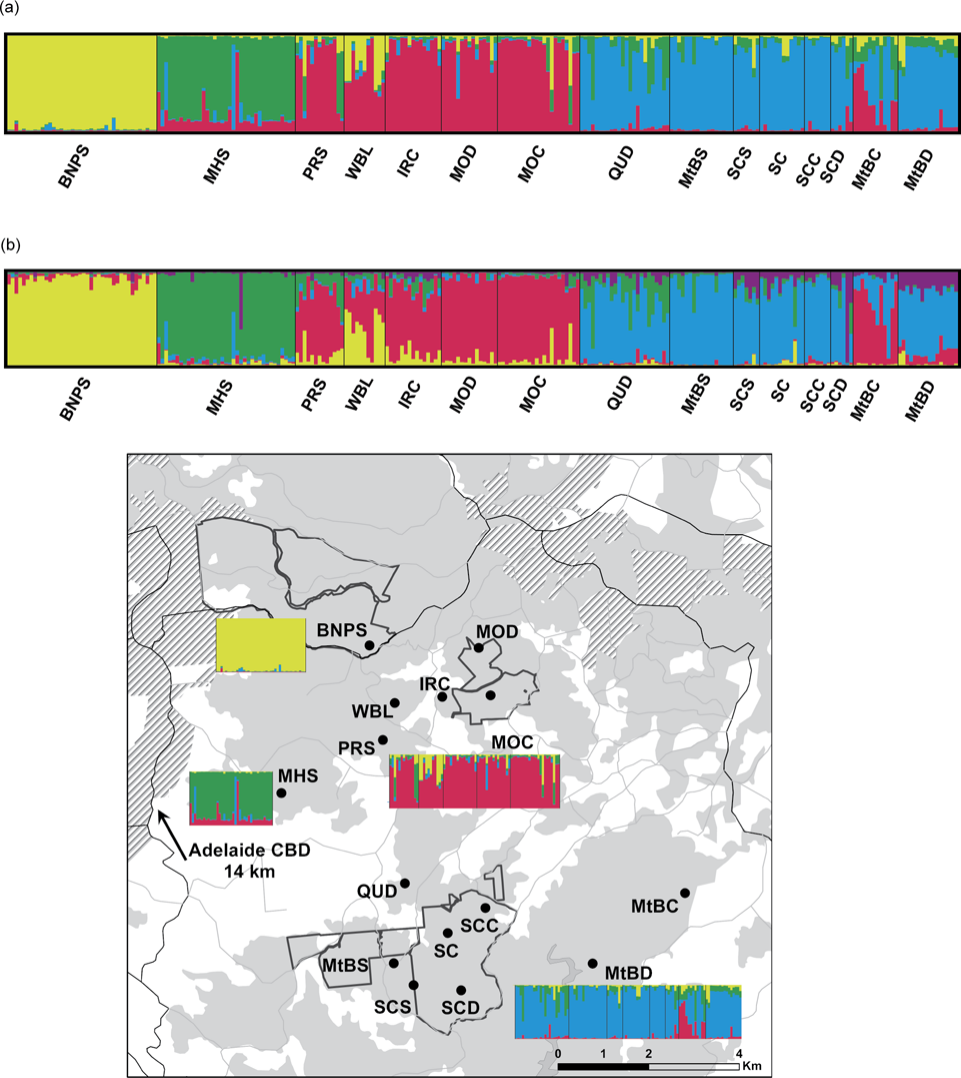
Genetic structure of bandicoots from 15 sites. Proportional membership (*q*) of each bandicoot individual to a genetic cluster for the whole data set, identified by: (a) STRUCTURE, (b) TESS. Each vertical bar represents a bandicoot, and the length of each bar represents the probability of membership in each cluster (cluster 1 in yellow, cluster 2 in green, cluster 3 in red and cluster 4 in blue). Relevant parts of the STRUCTURE plot are also shown on the map for better visualisation of locality information.

Using the program STRUCTURE, four clusters (*K* = 4) were identified (S1 Fig). Samples from BNPS grouped in one cluster (hereafter ‘BNPS cluster’), samples from MHS were distinct and grouped in a separate cluster (hereafter ‘MHS cluster’), PRS, WBL, IRC, MOC, MOD and MtBC in a third cluster (hereafter ‘northern cluster’), and QUD, MtBS, SCS, SC, SCC, SCD, and MtBD in a fourth cluster (hereafter ‘southern cluster’) (Fig 2A). Seventy-two percent of the individuals were assigned with a probability > 80% to one of the four clusters.

In the Bayesian clustering analysis computed by the program TESS, five clusters (*K* = 5) were identified, with four clusters being identical to that found using STRUCTURE (Fig 2B). The fifth cluster detected in TESS contained only two individuals and was not specific to any site, so was disregarded. We thus referred to *K* = 4 as the most likely number of genetic clusters in our dataset: the BNPS cluster, the MHS cluster, the northern cluster (PRS, WBL, IRC, MOC, MOD and MtBC) and the southern cluster (QUD, MtBS, SCS, SC, SCC, SCD, and MtBD) (Fig 2).

**Fig 3:**
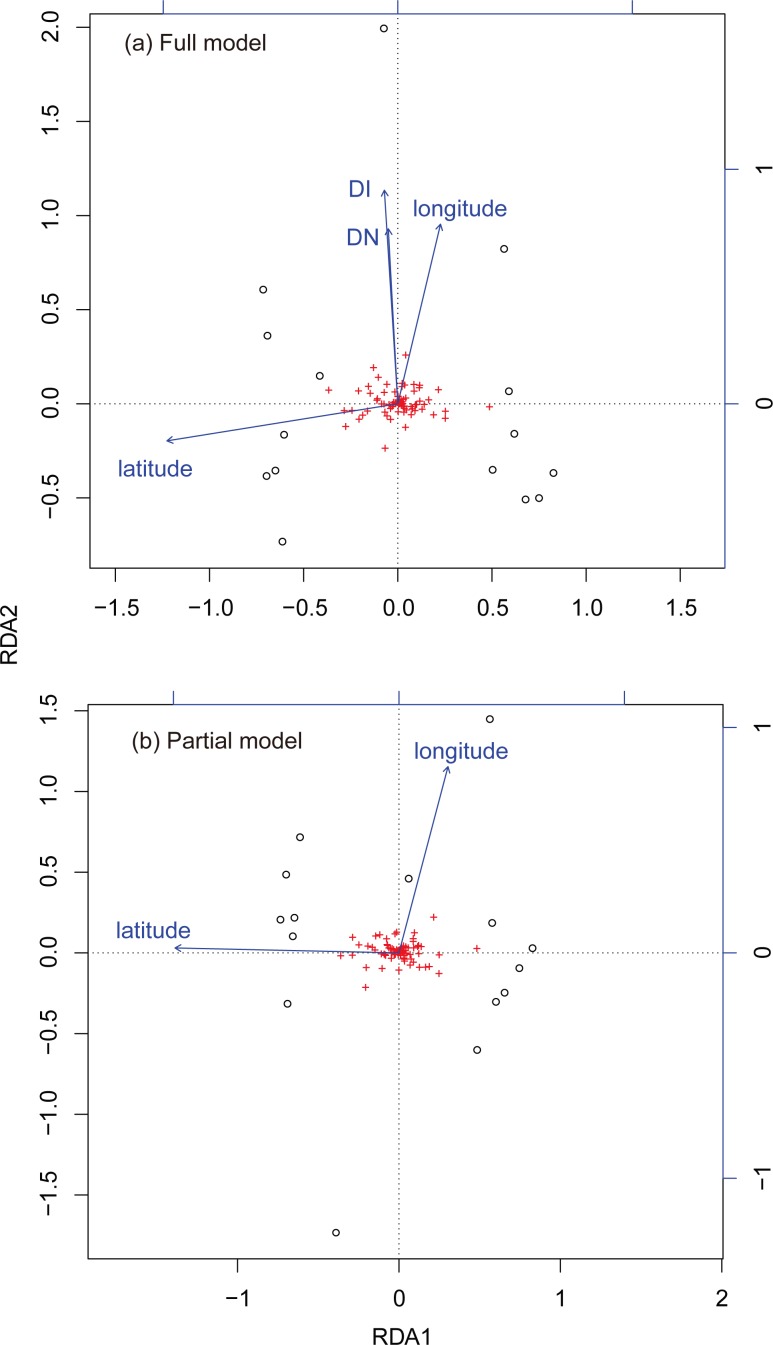
**Biplots of redundancy analysis (RDA) results showing the contribution of environmental components to genetic structure in *I*. *obesulus*, for (a) the full model and (b) the partial model.** The black (open) circles are allele frequencies of each site displayed in RDA space and the vectors show how explainable variables fall along that RDA space. DN = minimum distance to neighbouring patch; DI = degree of isolation.

RDA analysis with the full model showed a significant association between genetic variance and geographic location (*P* = 0.010, Fig 3A, showing the longest vector along each RDA axis). This relationship was also significant when the analysis was controlled for DN and DI (*P* = 0.005, Fig 3B). Partitioning of the variance components (comparing the full model with the partial models) indicated that geographic location explained 72.6% of the total explainable genetic variance when controlled for other variables; DN and DI explained 11.0% and 13.2% respectively; and all three variables taken together had a joint effect of 3.2% on genetic variance.

**Fig 4:**
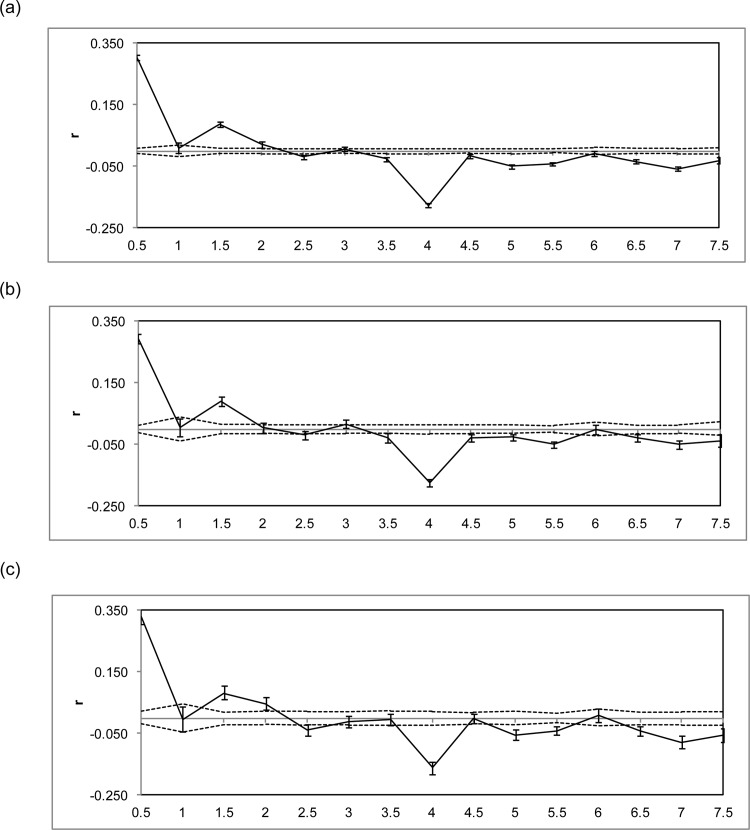
Correlograms showing genetic correlation (r) as a function of distance (0.5 km distance classes). The 95% confidence intervals (dashed lines) were determined by 1 000 permutations. Error bars of each estimate of r bound the 95% confidence intervals were determined by 1 000 bootstraps. (a) Whole data set; (b) Males only (n = 140) and (c) Females only (n = 105).

For the whole data set (males and females together), spatial autocorrelation analysis revealed a significant and positive correlation for individuals up to 1 km and the genetic similarities (r) then stabilized at a value around zero (Fig 4A). When analysed separately, the results for males and females were very similar (Fig 4B and 4C).

Bayesian estimation of migration rates from BayesAss indicated that total migration among STRUCTURE clusters was low and approximately equal in each direction, averaging 0.011 ± 0.007 (S2 Table). We found symmetric gene flow for most of the site pairs, with an average migration rate of 0.016 ± 0.016 (S3 Table). Migration for four site pairs were directionally biased: 0.142 from PRS to MOC and 0.009 from MOC to PRS; 0.116 from MOD to MOC and 0.018 from MOC to MOD; 0.042 from QUD to MtBS and 0.081 from MtBS to QUD; 0.021 from MtBS to SC and 0.100 from SC to MtBS (S3 Table).

A total of 22 individuals were identified as potential migrants (16 by STRUCTURE and 14 by GENECLASS, Table 3). Eight individuals were identified as migrants by both methods and were thus classified as such (Table 3). Of the remaining 14 individuals, three had a probability of >80% of belonging to their sampled sites in STRUCTURE and were thus classified as residents (Table 3). The remaining 11 individuals had *q*-values less than 0.8 and were thus classified as individuals with potentially mixed ancestry (Table 3) [60–63]. Seven of the eight migration events purportedly occurred between adjacent sites and one appeared to be an example of longer distance dispersal (indicative ~7 km).

**Table 3:** Results of migrants identified by STRUCTURE and GENECLASS analyses.

Sample ID	Sampled site	Sex	STRUCTURE probability to sampled site	GENECLASS *P* value	Final migrant/admixture/resident classification	Likely origin site in STRUCTURE/GENECLASS	Distance between origin site and sampled site* (km)
550	MHS	M	0.140	0.186	AD	-	-
564	MHS	M	0.075	0.102	AD	-	-
565	MHS	M	0.308	0.176	AD	-	-
595	WBL	M	0.117	<0.001	MG	MOC	2.1
501	IRC	M	0.229	0.065	AD	-	-
504	IRC	F	0.732	0.020	AD	-	-
833	MOD	F	0.947	0.023	RD	-	-
624	MOC	M	0.030	0.008	MG	PRS	1.1
736	MOC	M	0.413	0.706	AD	-	-
737	MOC	M	0.004	0.284	AD	-	-
742	MOC	M	0.004	0.016	MG	WBL	1.1
601	QUD	M	0.877	0.023	RD	-	-
675	QUD	F	0.235	0.004	MG	SC	1.4
645	MtBS	F	0.010	0.018	MG	SCC	2.4
648	MtBS	M	0.003	0.028	MG	SC	1.4
661	MtBS	M	<0.001	0.001	MG	SC	1.4
340	SC	M	0.304	0.051	AD	-	-
748	SCC	F	0.615	0.006	AD	-	-
757	SCD	M	0.025	0.961	AD	-	-
635	MtBC	M	0.800	0.023	RD	-	-
794	MtBC	F	<0.001	<0.001	MG	MtBS	6.6
785	MtBD	M	0.740	0.024	AD	-	-

MG = Migrant; AD = Admixture individual; RD = Resident

* The “distance between origin site and sampled site” was measured in ArcGIS 10 as straight-line distance.

## Discussion

In the current study, we used 14 microsatellite markers to investigate the population structure and level of dispersal of *I*. *obesulus* in the central Mount Lofty Ranges. We found significant genetic structure in a relatively small geographic region that is fragmented and highly modified. Overall, these analyses are consistent with the hypothesis that gene flow is severely limited to the extent that significant population genetic structure is evident at a fine spatial scale. They are also consistent with results obtained for a similar study from the south-east region of South Australia [26] (see Fig 1), although the current study provides evidence for an even finer level of population differentiation.

Bayesian clustering analyses revealed that the 15 Mount Lofty Ranges sites formed four distinct genetic clusters or populations. Analyses of migration rates also indicated an absence of recent migration between clusters and between most sites. In agreement with this, the eight first generation migrants detected were restricted to moving between neighbouring sites of the same population cluster, with one putative longer-distance dispersal which may have occurred via intermediate sites in a stepping-stone manner. Evidence for short distance dispersal and a tendency of dispersing only between proximate sites was also observed for *I*. *obesulus* in the Mount Burr (south-east South Australia) fragmented forest system [26] and for the common ringtail possum in the same region, where the pine matrix and cleared agricultural land were found to strongly influence dispersal of this arboreal species [17,64]. Short distance dispersal was also observed for a close relative of *I*. *obesulus*, the northern brown bandicoot (*I*. *macrourus*), in urban habitat fragments in Brisbane [65]. It is also a common feature of many other mammal (e.g. pikas, *Ochotona princeps* [66], cross river gorillas, *Gorilla gorilla diehli* [60], the edible dormouse *Glis glis* [67]) and vertebrate [68] species in fragmented landscapes, where dispersal is often limited to neighbouring patches and is strongly influenced by urban structures such as roads [68].

Scott Creek Conservation Park (712 ha, sites SCC, SC and SCD are located within this park) is one of the three national/conservation parks in our study area, but much larger than Mark Oliphant (189 ha, containing sites MOC and MOD). Compared to other sites, the three within Scott Creek Conservation Park showed non-significant pairwise *F*_*ST*_ (compared to other sites separated by equivalent geographic distances), lower individual relatedness, evidence for more migration events and directional migration to adjacent sites. These findings may reflect a larger population of bandicoots at each site, compared to other sites that we studied, hence genetic drift would occur more slowly, or it may suggest more extensive gene flow among each of the sites. Although we did not consider that Scott Creek CP was a continuous forest, because it contains areas of relatively open land, the park does contain extensive areas of thick vegetation that may have aided dispersal among sites and provided protection against predators. Large patches are considered important and critical in fragmented landscapes because they can reduce extinction proneness of populations of individual species, and increase species richness, vegetation diversity and immigration rates [12, 69]. Our results suggest that Scott Creek Conservation Park may be a source population for dispersal, although the importance of other smaller sites should not be underestimated since they are potential stepping stones between populations in fragmented systems. An ideal population genetic connectivity study would include a thorough comparison between continuous and fragmented habitat (e.g. [67, 70–71]). Unfortunately, information from a completely undisturbed habitat was not possible to obtain in the current study. However, the analyses of sites within Scott Creek CP highlights that the fine-scale population structure within *I*. *obesulus* evident from other sites in the Mount Lofty Ranges is most likely to have resulted from habitat fragmentation and the poor permeability of the matrix, rather than a natural feature of *I*. *obesulus* such as a poor dispersal ability. Further comparison of *I*. *obesulus* between isolated patches and relatively larger continuous forest would be beneficial and may be possible in other regions of Australia where continuous forest systems still occur (e.g Victoria, [72]).

Comparison of our results to those from south-east South Australia 400 km away [26], showed populations in the central Mount Lofty Ranges to be genetically structured over a much smaller spatial scale (~ 80 km^2^) than the south-east populations (~520 km^2^). We had predicted that the scale of genetic differentiation at Mount Lofty Ranges would be similar or even lower than that of Mount Burr since the former system is seemingly less fragmented. Yet, we observed that Mount Lofty Ranges sites appear to be genetically differentiated to a greater degree than the Mount Burr population at a similar spatial scale. This finding is consistent with the hypothesis that gene flow was limited to a higher degree in the Mount Lofty Ranges compared to Mount Burr, although we cannot rule out the possibility that the patterns resulted from differences in the effective population sizes of each study area. The landscape of the Mount Lofty Ranges has been heavily modified, with a matrix mixture of urban constructs and agricultural land and heterogeneous native vegetation with various level of degradation. The habitat within native forest fragments at Mount Burr is generally less disturbed and relatively more homogenous, with *Pinus radiata* plantations being the dominant matrix surrounding the fragments. The Mount Burr study suggested that bandicoot movement between proximate patches through pine plantations of a couple of km distance was possible. Hence, it is possible that bandicoots can utilise the pine forest better to move among native forest fragments, compared to the heterogeneous matrix mixture of the Mount Lofty Ranges. There are four potential reasons for this difference. First, pine plantations can be used as habitat by a range of invertebrate taxa, since they provide shelter and moist microhabitats due to plantation practices such as windrowing, mound ploughing, pruning and thinning [73, 74]. In particular, beetle species, a major dietary food for *I*. *obesulus* [75–77], have been found in pine plantations at greater levels of taxon diversity than in the native eucalypt forests [78]. Hence, bandicoots may occasionally enter pine plantations from adjacent patches for foraging. In a similar fashion, other insectivorous mammals may also forage in pine plantations from their home patch (e.g. *Antechinus agilis*, [79]). Second, accumulated fallen debris such as tree stumps and bark may provide better shelter for *I*. *obesulus* when dispersing in pine plantations, compared to open agricultural land. Third, the central Mount Lofty Ranges have greater vehicle traffic volumes than that in the Mount Burr Range (assessed based on Annual Average Daily Traffic estimates, produced by Road Asset Management Section, Government of South Australia), potentially leading to higher mortalities of *I*. *obesulus*. Deceased bandicoots along roads in the Mount Lofty Ranges have often been observed by local residents and staff of Department of Environment, Water, and Natural Resources (DEWNR). Another possible factor that could affect the dispersal of bandicoots in the two landscapes is predation by introduced animals (red fox, *Vulpes vulpes*, and feral cat, *Felis catus*). Studies have shown that the introduction of feral cats caused substantial wildlife mortality [80] and can extinguish an entire subspecies [81]. To date, it is not known whether the central Mount Lofty Ranges have more predators than the Mount Burr Range, though cats, in particular, are likely to be more prevalent given the higher number of dwellings and human population density in the Mount Lofty Ranges compared to the Mount Burr Range. More sophisticated analyses on the same dataset (i.e. using landscape genetic approaches, [82]) are needed to further investigate how different features of the matrices affect genetic connectivity of *I*. *obesulus* populations.

The apparent poor permeability of the matrix in the Mount Lofty Ranges may also explain the strong differentiation of the most genetically isolated site—BNPS. This site also had the highest pairwise *F*_*ST*_ and *D*_*EST*_ and the highest internal relatedness, suggesting that inbreeding may be occurring. Major roads to the south and north with high traffic volumes are adjacent to BNPS and may also impact dispersal. Road effects on increased genetic differentiation and decreased genetic connectivity have been reported in an increasing array of species, including mammals, amphibians, reptiles and invertebrates (e.g. [4, 20, 83–88], and see also reviews of [89–92]). Notably, adjacent sites (e.g. WBL and PRS) did show some evidence of admixture with BNPS, but there is no evidence of admixture within BNPS itself (see Fig 2). In agreement with this, migration rates from BNPS to WBL and PRS were 0.012 and 0.025, respectively, and 0.006 from WBL/PRS to BNPS (S3 Table). These results suggest that gene flow may be occurring in only one direction (from BNPS to adjacent sites). While these results may have been influenced by the related individuals at BNPS, excluding related individuals did not alter the results showing the uni-directional gene flow. One possible reason for the above pattern is that there is now a failure of recruitment of young animals into the Belair NP population (e.g. by predation of young by cats) or a recent reduction in suitable habitat leading to a smaller effective population size within the park and/or migration out of the park. Further ecological and genetic studies of the BNPS population are needed to investigate these possibilities.

### Implications for conservation

Comparisons among populations of the same species can help assess the levels of permeability of different matrices (e.g. [20, 21, 93]), which may provide valuable information for conservation management (e.g. developing strategies to improve gene flow in the landscape). The construction of habitat corridors is one widely used approach to promote population connectivity in fragmented landscapes and it is the primary conservation management action plan for the Mount Burr population [94]. It may also benefit connectivity between sites in the Mount Lofty Ranges, but this approach may be impractical in some areas in the latter system due to the embedded human constructs. Retention and restoration of native vegetation to improve and extend suitable bandicoot habitat is therefore recommended as a complementary way to deal with habitat fragmentation in this region. Thick exotic vegetation, such as blackberries (*Rubus fruticosus* agg), along creek lines may also assist dispersal of bandicoots, and its removal, without replacement of a dense understory vegetation is likely to further fragment bandicoot populations [95].

In order to reduce the effects of inbreeding and increase long-term persistence of the numerous genetically distinct populations, the management of these populations may benefit from augmentation of gene flow between populations. Such genetic rescue and/or genetic restoration could be accomplished by moving individuals between disparate populations (e.g. moving individuals into BNPS and MHS from their adjacent sites). If translocations were considered, we suggest managers should first evaluate the risks associated with translocations and consider potential mitigation strategies, as recommended by Weeks et al. [96]. If bandicoots were sourced from other regions of the Mount Lofty Ranges, we would predict that outbreeding depression would be unlikely (given evidence for admixture in adjacent sites to BNPS). However, our recent phylogeographic analyses [97] suggest that the population in the south-east of SA represents a distinct Evolutionarily Significant Unit compared to the Mount Lofty Ranges population, so use of south-east SA populations as a source of bandicoots for translocation should probably be avoided. Our results indicate that site BNPS is more vulnerable to local extinction and therefore should be managed with a specific strategy that enhances genetic variation. To further ameliorate the effects of the major road on site BNPS, mitigation measures such as wildlife crossing structures (e.g. land bridge, ledges in culvert, underground tunnel or pipe) could also be implemented. Such structures are known to be utilised by animals for crossing roads, but their effectiveness in mitigating the influence of roads on gene flow, population structure and ultimately population viability remains to be determined [98] (but see [99]). However, our study provides valuable base-line genetic data which can be used in an optimal before-after study design for assessing the effectiveness of mitigation strategies in the future [100].

## Supporting Information

S1 Fig Plot of the number of likely clusters (*K*) versus estimated Ln of probability of data. (DOCX)

S1 Table Results for post hoc tests (Tukey HSD) of *IR* for *I*. *obesulus* at 15 sites within the Mount Lofty Ranges. Significant values were denoted in bold (*P* < 0.05).
(DOCX)

S2 Table Bayesian estimates of migration rates in BayesAss among genetic clusters. Migration rates greater than 2% are shown in bold, and self-migration rates shown in italics. Standard deviation of migration rates averaged 0.010 and did not exceed 0.022 (BNPS cluster-BNPS cluster).
(DOCX)

S3 Table Bayesian estimates of migration rates in BayesAss among 15 sites. Migration rates greater than 2% are shown in bold, and self-migration rates shown in italics. Standard deviation of migration rates averaged 0.014 and did not exceed 0.053 (SCC-SCS).
(DOCX)
