# Supplementary material for: Comparative Population Genetic Structure of the Endangered Southern Brown Bandicoot, Isoodon obesulus, in Fragmented Landscapes of Southern Australia
Source: PLoS One. 2016 Apr 20;11(4):e0152850. doi: 10.1371/journal.pone.0152850 (PMC4838232; doi:10.1371/journal.pone.0152850)
Supplement: S1 Fig — (DOCX) [file pone.0152850.s001.docx]

**Supporting Information**

**S1 Fig**

**

**

**S1 Fig. Plot of the number of likely clusters (*K*) versus estimated Ln of probability of data.**
