# Supplementary material for: Comparative Population Genetic Structure of the Endangered Southern Brown Bandicoot, Isoodon obesulus, in Fragmented Landscapes of Southern Australia
Source: PLoS One. 2016 Apr 20;11(4):e0152850. doi: 10.1371/journal.pone.0152850 (PMC4838232; doi:10.1371/journal.pone.0152850)
Supplement: S1 Table — Significant values were denoted in bold (P < 0.05). (DOCX) [file pone.0152850.s002.docx]

**Supporting Information**

**S1 Table**

**S1 Table.** **Results for post hoc tests (Tukey HSD) of *IR* for *I. obesulus* at 15 sites within the Mount Lofty Ranges.** Significant values were denoted in bold (*P* < 0.05).

| *IR* | | Std. Error | *P* value |
| --- | --- | --- | --- |
|  |  |  |  |
| BNPS | IRC | .065103 | .451 |
|  | MHS | .049047 | **.000** |
|  | MOC | .057076 | **.004** |
|  | MOD | .065103 | **.001** |
|  | MtBC | .055520 | **.016** |
|  | MtBD | .050158 | **.000** |
|  | MtBS | .062256 | .774 |
|  | PRS | .068649 | .180 |
|  | QUD | .055520 | **.001** |
|  | SC | .070775 | .998 |
|  | SCC | .088098 | **.000** |
|  | SCD | .094139 | **.009** |
|  | SCS | .088098 | **.000** |
|  | WBL | .073208 | **.009** |
| IRC | BNPS | .065103 | .451 |
|  | MHS | .065819 | .999 |
|  | MOC | .072001 | .999 |
|  | MOD | .078517 | .925 |
|  | MtBC | .070775 | 1.000 |
|  | MtBD | .066651 | .937 |
|  | MtBS | .076173 | 1.000 |
|  | PRS | .081481 | 1.000 |
|  | QUD | .070775 | .996 |
|  | SC | .083280 | 1.000 |
|  | SCC | .098427 | .002 |
|  | SCD | .103869 | .797 |
|  | SCS | .098427 | .348 |
|  | WBL | .085357 | .980 |
| MHS | BNPS | .049047 | **.000** |
|  | IRC | .065819 | .999 |
|  | MOC | .057891 | 1.000 |
|  | MOD | .065819 | 1.000 |
|  | MtBC | .056358 | 1.000 |
|  | MtBD | .051084 | 1.000 |
|  | MtBS | .063004 | .919 |
|  | PRS | .069328 | 1.000 |
|  | QUD | .056358 | 1.000 |
|  | SC | .071434 | .705 |
|  | SCC | .088628 | **.008** |
|  | SCD | .094636 | .986 |
|  | SCS | .088628 | .737 |
|  | WBL | .073845 | 1.000 |
| MOC | BNPS | .057076 | **.004** |
|  | IRC | .072001 | .999 |
|  | MHS | .057891 | 1.000 |
|  | MOD | .072001 | 1.000 |
|  | MtBC | .063469 | 1.000 |
|  | MtBD | .058836 | 1.000 |
|  | MtBS | .069437 | .957 |
|  | PRS | .075223 | 1.000 |
|  | QUD | .063469 | 1.000 |
|  | SC | .077167 | .793 |
|  | SCC | .093312 | **.017** |
|  | SCD | .099035 | .992 |
|  | SCS | .093312 | .813 |
|  | WBL | .079405 | 1.000 |
| MOD | BNPS | .065103 | **.001** |
|  | IRC | .078517 | .925 |
|  | MHS | .065819 | 1.000 |
|  | MOC | .072001 | 1.000 |
|  | MtBC | .070775 | .996 |
|  | MtBD | .066651 | 1.000 |
|  | MtBS | .076173 | .638 |
|  | PRS | .081481 | .998 |
|  | QUD | .070775 | 1.000 |
|  | SC | .083280 | .396 |
|  | SCC | .098427 | .177 |
|  | SCD | .103869 | 1.000 |
|  | SCS | .098427 | .995 |
|  | WBL | .085357 | 1.000 |
| MtBC | BNPS | .055520 | **.016** |
|  | IRC | .070775 | 1.000 |
|  | MHS | .056358 | 1.000 |
|  | MOC | .063469 | 1.000 |
|  | MOD | .070775 | .996 |
|  | MtBD | .057328 | .998 |
|  | MtBS | .068165 | .997 |
|  | PRS | .074049 | 1.000 |
|  | QUD | .062074 | 1.000 |
|  | SC | .076024 | .944 |
|  | SCC | .092368 | **.005** |
|  | SCD | .098147 | .949 |
|  | SCS | .092368 | .588 |
|  | WBL | .078294 | 1.000 |
| MtBD | BNPS | .050158 | **.000** |
|  | IRC | .066651 | .937 |
|  | MHS | .051084 | 1.000 |
|  | MOC | .058836 | 1.000 |
|  | MOD | .066651 | 1.000 |
|  | MtBC | .057328 | .998 |
|  | MtBS | .063873 | .586 |
|  | PRS | .070119 | 1.000 |
|  | QUD | .057328 | 1.000 |
|  | SC | .072201 | .346 |
|  | SCC | .089248 | **.037** |
|  | SCD | .095216 | 1.000 |
|  | SCS | .089248 | .944 |
|  | WBL | .074588 | 1.000 |
| MtBS | BNPS | .062256 | .774 |
|  | IRC | .076173 | 1.000 |
|  | MHS | .063004 | .919 |
|  | MOC | .069437 | .957 |
|  | MOD | .076173 | .638 |
|  | MtBC | .068165 | .997 |
|  | MtBD | .063873 | .586 |
|  | PRS | .079225 | 1.000 |
|  | QUD | .068165 | .890 |
|  | SC | .081074 | 1.000 |
|  | SCC | .096567 | **.000** |
|  | SCD | .102108 | .531 |
|  | SCS | .096567 | .139 |
|  | WBL | .083206 | .836 |
| PRS | BNPS | .068649 | .180 |
|  | IRC | .081481 | 1.000 |
|  | MHS | .069328 | 1.000 |
|  | MOC | .075223 | 1.000 |
|  | MOD | .081481 | .998 |
|  | MtBC | .074049 | 1.000 |
|  | MtBD | .070119 | 1.000 |
|  | MtBS | .079225 | 1.000 |
|  | QUD | .074049 | 1.000 |
|  | SC | .086080 | .988 |
|  | SCC | .100807 | **.013** |
|  | SCD | .106127 | .963 |
|  | SCS | .100807 | .681 |
|  | WBL | .088092 | 1.000 |
| QUD | BNPS | .055520 | **.001** |
|  | IRC | .070775 | .996 |
|  | MHS | .056358 | 1.000 |
|  | MOC | .063469 | 1.000 |
|  | MOD | .070775 | 1.000 |
|  | MtBC | .062074 | 1.000 |
|  | MtBD | .057328 | 1.000 |
|  | MtBS | .068165 | .890 |
|  | PRS | .074049 | 1.000 |
|  | SC | .076024 | .664 |
|  | SCC | .092368 | **.024** |
|  | SCD | .098147 | .997 |
|  | SCS | .092368 | .872 |
|  | WBL | .078294 | 1.000 |
| SC | BNPS | .070775 | .998 |
|  | IRC | .083280 | 1.000 |
|  | MHS | .071434 | .705 |
|  | MOC | .077167 | .793 |
|  | MOD | .083280 | .396 |
|  | MtBC | .076024 | .944 |
|  | MtBD | .072201 | .346 |
|  | MtBS | .081074 | 1.000 |
|  | PRS | .086080 | .988 |
|  | QUD | .076024 | .664 |
|  | SCC | .102267 | **.000** |
|  | SCD | .107514 | .332 |
|  | SCS | .102267 | .069 |
|  | WBL | .089758 | .611 |
| SCC | BNPS | .088098 | **.000** |
|  | IRC | .098427 | **.002** |
|  | MHS | .088628 | **.008** |
|  | MOC | .093312 | **.017** |
|  | MOD | .098427 | .177 |
|  | MtBC | .092368 | **.005** |
|  | MtBD | .089248 | **.037** |
|  | MtBS | .096567 | **.000** |
|  | PRS | .100807 | **.013** |
|  | QUD | .092368 | **.024** |
|  | SC | .102267 | **.000** |
|  | SCD | .119631 | .886 |
|  | SCS | .114938 | .982 |
|  | WBL | .103965 | .206 |
| SCD | BNPS | .094139 | **.009** |
|  | IRC | .103869 | .797 |
|  | MHS | .094636 | .986 |
|  | MOC | .099035 | .992 |
|  | MOD | .103869 | 1.000 |
|  | MtBC | .098147 | .949 |
|  | MtBD | .095216 | 1.000 |
|  | MtBS | .102108 | .531 |
|  | PRS | .106127 | .963 |
|  | QUD | .098147 | .997 |
|  | SC | .107514 | .332 |
|  | SCC | .119631 | .886 |
|  | SCS | .119631 | 1.000 |
|  | WBL | .109131 | 1.000 |
| SCS | BNPS | .088098 | **.000** |
|  | IRC | .098427 | .348 |
|  | MHS | .088628 | .737 |
|  | MOC | .093312 | .813 |
|  | MOD | .098427 | .995 |
|  | MtBC | .092368 | .588 |
|  | MtBD | .089248 | .944 |
|  | MtBS | .096567 | .139 |
|  | PRS | .100807 | .681 |
|  | QUD | .092368 | .872 |
|  | SC | .102267 | .069 |
|  | SCC | .114938 | .982 |
|  | SCD | .119631 | 1.000 |
|  | WBL | .103965 | .994 |
| WBL | BNPS | .073208 | **.009** |
|  | IRC | .085357 | .980 |
|  | MHS | .073845 | 1.000 |
|  | MOC | .079405 | 1.000 |
|  | MOD | .085357 | 1.000 |
|  | MtBC | .078294 | 1.000 |
|  | MtBD | .074588 | 1.000 |
|  | MtBS | .083206 | .836 |
|  | PRS | .088092 | 1.000 |
|  | QUD | .078294 | 1.000 |
|  | SC | .089758 | .611 |
|  | SCC | .103965 | .206 |
|  | SCD | .109131 | 1.000 |
|  | SCS | .103965 | .994 |
