# Supplementary material for: Comparative Population Genetic Structure of the Endangered Southern Brown Bandicoot, Isoodon obesulus, in Fragmented Landscapes of Southern Australia
Source: PLoS One. 2016 Apr 20;11(4):e0152850. doi: 10.1371/journal.pone.0152850 (PMC4838232; doi:10.1371/journal.pone.0152850)
Supplement: S2 Table — Migration rates greater than 2% are shown in bold, and self-migration rates shown in italics. Standard deviation of migration rates averaged 0.010 and did not exceed 0.022 (BNPS cluster-BNPS cluster). (DOCX) [file pone.0152850.s003.docx]

**Supporting Information**

**S2 Table**

**S2 Table.** **Bayesian estimates of migration rates in BayesAss among genetic clusters.** Migration rates greater than 2% are shown in bold, and self-migration rates shown in italics. Standard deviation of migration rates averaged 0.010 and did not exceed 0.022 (BNPS cluster-BNPS cluster).

| From | To | | | |
| --- | --- | --- | --- | --- |
|  | BNPS cluster | MHS cluster | northern cluster | southern cluster |
| BNPS cluster | *0.976* | 0.008 | 0.012 | 0.005 |
| MHS cluster | 0.008 | *0.945* | 0.005 | 0.011 |
| northern cluster | 0.009 | 0.018 | *0.968* | 0.008 |
| southern cluster | 0.008 | **0.028** | 0.016 | *0.976* |
