# Supplementary material for: Comparative Population Genetic Structure of the Endangered Southern Brown Bandicoot, Isoodon obesulus, in Fragmented Landscapes of Southern Australia
Source: PLoS One. 2016 Apr 20;11(4):e0152850. doi: 10.1371/journal.pone.0152850 (PMC4838232; doi:10.1371/journal.pone.0152850)
Supplement: S3 Table — Migration rates greater than 2% are shown in bold, and self-migration rates shown in italics. Standard deviation of migration rates averaged 0.014 and did not exceed 0.053 (SCC-SCS). (DOCX) [file pone.0152850.s004.docx]

**Supporting Information**

**S3 Table**

| From | To | | | | | | | | | | | | | | |
| --- | --- | --- | --- | --- | --- | --- | --- | --- | --- | --- | --- | --- | --- | --- | --- |
|  | BNPS | MHS | PRS | WBL | IRC | MOD | MOC | QUD | MtBS | SCS | SC | SCC | SCD | MtBC | MtBD |
| BNPS | *0.919* | 0.006 | 0.012 | **0.025** | 0.011 | 0.011 | 0.011 | 0.010 | 0.010 | 0.015 | 0.014 | 0.015 | 0.016 | 0.012 | 0.011 |
| MHS | 0.006 | *0.868* | 0.013 | 0.012 | 0.011 | 0.015 | 0.013 | 0.011 | 0.011 | 0.018 | 0.014 | 0.016 | 0.018 | 0.013 | 0.011 |
| PRS | 0.006 | 0.006 | *0.679* | 0.013 | 0.011 | 0.011 | **0.142** | 0.009 | 0.010 | 0.015 | 0.012 | 0.015 | 0.019 | 0.012 | 0.011 |
| WBL | 0.006 | 0.006 | **0.031** | *0.773* | **0.040** | 0.018 | **0.021** | 0.009 | 0.010 | 0.017 | 0.013 | 0.015 | 0.017 | 0.013 | 0.011 |
| IRC | 0.006 | 0.007 | 0.013 | 0.015 | *0.797* | 0.015 | 0.010 | 0.010 | 0.011 | 0.015 | 0.012 | 0.015 | 0.017 | 0.012 | 0.011 |
| MOD | 0.006 | 0.016 | 0.013 | 0.016 | 0.018 | *0.809* | **0.116** | 0.009 | 0.011 | 0.016 | 0.016 | 0.016 | 0.017 | 0.013 | 0.011 |
| MOC | 0.006 | 0.010 | 0.009 | **0.023** | **0.021** | 0.018 | *0.744* | 0.014 | 0.010 | 0.016 | 0.013 | 0.015 | 0.017 | 0.015 | 0.011 |
| QUD | 0.006 | **0.022** | 0.012 | 0.013 | 0.012 | 0.015 | 0.011 | *0.769* | **0.042** | **0.024** | 0.014 | **0.020** | **0.034** | 0.017 | 0.012 |
| MtBS | 0.006 | 0.011 | 0.012 | **0.030** | 0.012 | 0.020 | 0.009 | **0.081** | *0.805* | **0.027** | **0.021** | 0.016 | 0.018 | **0.031** | 0.012 |
| SCS | 0.006 | 0.007 | 0.012 | 0.013 | 0.011 | 0.011 | 0.009 | 0.010 | 0.011 | *0.701* | 0.014 | 0.016 | **0.030** | 0.013 | **0.022** |
| SC | 0.006 | 0.007 | 0.012 | 0.017 | 0.011 | 0.012 | 0.009 | 0.018 | **0.100** | 0.018 | *0.757* | **0.020** | 0.020 | 0.013 | 0.011 |
| SCC | 0.006 | 0.013 | 0.012 | 0.013 | 0.011 | 0.011 | 0.009 | 0.019 | **0.037** | **0.070** | **0.046** | *0.687* | 0.060 | 0.012 | **0.021** |
| SCD | 0.006 | 0.007 | 0.012 | 0.013 | 0.011 | 0.011 | 0.009 | 0.009 | 0.011 | 0.017 | 0.013 | 0.016 | *0.684* | 0.012 | 0.011 |
| MtBC | 0.006 | 0.007 | 0.014 | 0.013 | 0.011 | 0.013 | 0.011 | 0.010 | 0.011 | 0.016 | 0.013 | 0.017 | 0.017 | *0.798* | 0.013 |
| MtBD | 0.006 | 0.007 | 0.012 | 0.013 | 0.011 | 0.011 | 0.009 | 0.012 | 0.011 | 0.017 | 0.012 | 0.016 | 0.018 | 0.013 | *0.822* |

**S3 Table.** **Bayesian estimates of migration rates in BayesAss among 15 sites.** Migration rates greater than 2% are shown in bold, and self-migration rates shown in italics. Standard deviation of migration rates averaged 0.014 and did not exceed 0.053 (SCC-SCS).
